# Exploring water-borne corticosterone collection as a non-invasive tool in amphibian conservation physiology: benefits, limitations and future perspectives

**DOI:** 10.1093/conphys/coad070

**Published:** 2023-09-01

**Authors:** Katharina Ruthsatz, Rafael Rico-Millan, Paula Cabral Eterovick, Ivan Gomez-Mestre

**Affiliations:** Zoological Institute, Technische Universität Braunschweig, Mendelssohnstraße 4, 38106 Braunschweig, Germany; Ecology, Evolution, and Development Group, Doñana Biological Station – CSIC, Calle Américo Vespucio 26, 41092 Seville, Spain; Zoological Institute, Technische Universität Braunschweig, Mendelssohnstraße 4, 38106 Braunschweig, Germany; Ecology, Evolution, and Development Group, Doñana Biological Station – CSIC, Calle Américo Vespucio 26, 41092 Seville, Spain

**Keywords:** Biomarker, conservation physiology toolbox, environmental stress, European common frog/Rana temporaria, glucocorticoid hormone, metamorphosis

## Abstract

Global change exposes wildlife to a variety of environmental stressors and is affecting biodiversity worldwide, with amphibian population declines being at the forefront of the global biodiversity crisis. The use of non-invasive methods to determine the physiological state in response to environmental stressors is therefore an important advance in the field of conservation physiology. The glucocorticoid hormone corticosterone (CORT) is one useful biomarker to assess physiological stress in amphibians, and sampling water-borne (WB) CORT is a novel, non-invasive collection technique. Here, we tested whether WB CORT can serve as a valid proxy of organismal levels of CORT in larvae of the common frog (*Rana temporaria*). We evaluated the association between tissue and WB CORT levels sampled from the same individuals across ontogenetic stages, ranging from newly hatched larvae to froglets at 10 days after metamorphosis. We also investigated how both tissue and WB CORT change throughout ontogeny. We found that WB CORT is a valid method in pro-metamorphic larvae as values for both methods were highly correlated. In contrast, there was no correlation between tissue and WB CORT in newly hatched, pre-metamorphic larvae, metamorphs or post-metamorphic froglets probably due to ontogenetic changes in respiratory and skin morphology and physiology affecting the transdermal CORT release. Both collection methods consistently revealed a non-linear pattern of ontogenetic change in CORT with a peak at metamorphic climax. Thus, our results indicate that WB CORT sampling is a promising, non-invasive conservation tool for studies on late-stage amphibian larvae. However, we suggest considering that different contexts might affect the reliability of WB CORT and consequently urge future studies to validate this method whenever it is used in new approaches. We conclude proposing some recommendations and perspectives on the use of WB CORT that will aid in broadening its application as a non-invasive tool in amphibian conservation physiology.

## Teaser

Across ontogeny, we tested whether water-borne corticosterone can serve as a valid representation of corticosterone concentration in the tissues of larvae of the common frog (Rana temporaria), and found this sampling method to be a promising non-invasive conservation tool for studies on late-stage amphibian larvae.

## Introduction

Glucocorticoid hormones (GCs) are metabolic hormones secreted from the hypothalamic–pituitary–adrenal/interrenal axis in vertebrates (Denver, 2009) that play an important role in animal growth and development, energy allocation, immune functioning, behaviour and in maintaining homeostasis (Romero, 2004; Crespi *et al.,* 2013; Kirschman *et al.,* 2017; Rollins-Smith, 2017). GCs are further associated with the physiological stress response (Sapolsky, 2002) and thus, measurements of the main GCs cortisol and/or corticosterone are one useful tool to determine the physiological state of animals in response to environmental stressors (Sapolsky *et al.,* 2000; Dantzer *et al.,* 2014). When homeostasis is disrupted, the central nervous system activates the hypothalamic–pituitary–adrenal/interrenal axis (Sapolsky, 2002; Dantzer *et al.,* 2014), resulting in the release of corticotropin-releasing factor from the hypothalamus that, in turn, activates the release of adrenocorticotropin from the pituitary (Denver, 1998). The latter induces the production of the GCs cortisol and/or corticosterone in the adrenal (cortisol) or interrenal (corticosterone) glands (Romero and Butler, 2007). Whereas cortisol is the predominant GC in most primates, ungulates, and teleost fish (Schreck and Tort, 2016; Suarez-Bregua *et al.,* 2018), corticosterone (CORT) is the major GC in sauropsids (Angelier and Chastel, 2009) and amphibians (Glennemeier and Denver, 2002; Denver, 2009; but not neotenic *Cryptobranchus alleganiensis,*Hopkins *et al.*, 2020 and larval and adult *Lithobates catesbeinus,*Wright *et al.*, 2003). GC levels can be obtained from a variety of sample media that differ in invasiveness and duration of handling required (Madliger *et al.,* 2018), ranging from completely invasive (e.g. whole-body homogenates/tissue), mildly invasive (e.g. blood drawing, saliva/skin swabbing), low invasiveness (e.g. obtaining hair/feather samples), to non-invasive (e.g. passive urine/faeces collection) (rev. in Sheriff *et al.,* 2011). In aquatic vertebrates, water-borne (WB) hormone sampling is a novel, non-invasive collection technique (fish: rev. in Scott and Ellis, 2007; amphibians: Gabor *et al.,* 2013a) that is highly repeatable (Forsburg *et al.,* 2019). GCs can pass through gills, mucous membranes and skin into the surrounding water (rev. in McClelland and Woodley, 2021). The water in which the specimen has been contained for 1–2 hours is collected and GCs are extracted from the water (Scott and Ellis, 2007; Gabor *et al.,* 2013a) and measured using immunoassays (Burraco *et al.,* 2015).

The use of non-invasive methods such as WB hormone sampling is an important advance in the field of conservation physiology (Narayan *et al.,* 2019) as it allows for repeated measurements across time and life history, reduces handling time, does not require harming or sacrificing individuals and thus constitutes a great tool in conservation biology (Madliger *et al.,* 2018). This is of key importance for assessing physiological stress in amphibian populations before they begin to decline as a consequence of the various environmental stressors associated with anthropogenic global change (Stuart *et al.,* 2004; IUCN, 2023).

However, WB CORT has been suggested to be a less sensitive biomarker for assessing the effect of environmental stress (Ruthsatz *et al.,* 2023) than other sampling methods, as the release of CORT into the water is likely influenced by several factors such as developmental stage, individual variability and rearing conditions (rev. in Tornabene *et al.,* 2021). This is particularly important for amphibian larvae, as endogenous CORT levels change throughout ontogeny (Glennemeier and Denver, 2002; Chambers *et al.,* 2011; Kulkarni and Buchholz, 2014) and life stage-specific CORT release might be impacted by ontogenetic changes in physiology (e.g. gill degeneration; Burggren and West, 1982) and morphology (e.g. increasing keratinization; Shi, 2000; rev. in Schreiber and Brown, 2003) that affect the surface for CORT release.

In this study, we evaluated whether WB CORT can serve as a valid representative of organismal levels of CORT in the common frog (*Rana temporaria*), a common model species for testing the effect of environmental variation on amphibians at different life stages under both field and laboratory conditions. Specifically, we measured WB and tissue CORT at different ontogenetic stages ranging from newly hatched larvae to froglets at ten days after completing metamorphosis. We predicted that both sampling methods would reveal an ontogenetic change in CORT with a peak at metamorphic climax (Denver, 1998; Chambers *et al.,* 2011; Regueira *et al.,* 2022) and that tissue and WB CORT within individuals would be highly associated (McClelland and Woodley, 2021). Considering the various approaches and contexts in which this non-invasive method might be used that might affect its reliability, we proposed some recommendations and perspectives on the use of WB CORT in amphibian conservation physiology.

## Materials and Methods

### Ethics statement

The experiments were conducted under permission from the *Niedersächsisches Landesamt für Verbraucherschutz und Lebensmittelsicherheit*, Germany (Gz. 33.19-42502-04-20/3590). Fieldwork in Lower Saxony was carried out with permits of Stadt Braunschweig (Stadt Braunschweig-Fachbereich Umwelt und Naturschutz, Richard-Wagner-Straße 1, 38 106 Braunschweig; Gz. 68.11-11.8-3.3).

### Experimental setup and animal husbandry

Six clutches of the European common frog (*R. temporaria*) were collected at Kleiwiesen (52.328 N, 10.582 E), a locality in central Germany near Braunschweig, Lower Saxony. We brought the eggs back to the laboratory at the Zoological Institute at Technische Universität Braunschweig, and conducted the experiment in a climatic chamber (Kälte-Klimatechnik-Frauenstein GmbH, Germany) with a 14:10 h light:dark cycle. Eggs were kept until hatching in six 12-L plastic buckets filled with 5 L of aged, dechlorinated tap water at 14 ± 0.2°C. After hatching, five larvae from each clutch were randomly selected and allocated to 6 standard 18-L aquaria filled with 16 L of aged, dechlorinated water. Each aquarium housed 30 larvae (30 larvae × 4 aquaria = 120 larvae in total). Aquaria were kept at 20 ± 0.1°C. Temperatures were chosen to reflect what larvae experience in their natural habitat. When larvae were approaching the onset of metamorphosis, tanks were surveyed daily to retrieve metamorphosing individuals (i.e. at forelimb emergence, Gosner stage 42; Gosner, 1960). Metamorphs were removed from aquaria and kept individually in 470 mL lidded plastic containers with 2 mm of aged de-chlorinated water at the bottom until they completed tail resorption (Gosner stage 46). After completing metamorphosis, the water in the plastic containers was removed and replaced by a wet paper towel, which was renewed daily.

Larvae were fed 50% high-protein powdered fish food (Sera micron breeding food for fish and amphibians, Germany) and 50% spirulina algae. *Ad libitum* rations were provided twice a day to guarantee that food was available in abundance. After completing metamorphosis, froglets were fed *ad libitum* with adult *Drosophila melanogaster* for 10 days prior to being subjected to the final measurements. The experiments ran for 8 weeks. All survivors not selected for final measurements were then released at their ponds of origin.

### Ontogenetic staging and sampling

We determined ontogenetic stages evaluating the progression of key morphological features during development, as detailed in Gosner (1960). To determine developmental stage, individuals were visually inspected one day prior to sampling with bare eye or examined under a binocular microscope (Keyence X-500).

We randomly collected two larvae from each of the four aquaria at eight ontogenetic stages (Gosner stage 20, 25, 30, 37, 39, 42, 46 and 46 + 10 days) covering from newly hatched larvae to juvenile froglets at 10 days after completing metamorphosis (i.e. 8 specimens per ontogenetic stage, 64 in total). Animals were collected before the regular water change with a small metal-made kitchen strainer (tadpoles: Gosner stage 20, 25, 30, 37, 39, and 42) or a tablespoon (metamorphs and juveniles: Gosner stage 46 and 46 + 10 days).

As animals reached the target developmental stages, we proceeded to WB CORT collection and subsequently euthanized them for tissue CORT measurements.

#### Water-borne CORT

To determine stage-specific CORT release into holding water, we used the established WB assay protocol by Gabor *et al.* (2013a). Briefly, each animal was gently placed in a freshly cleaned (1x EtOH, 3 x water) 250 mL glass beakers containing 25 mL (Gosner stage 20, 25, 46, and 46 + 10 days) or 50 mL (Gosner stage 30, 37, 39, and 42) of aged, filtered and previously aerated tap water for 1 h after collection. Glass beakers were placed in a water bath to maintain a constant temperature. A maximum of 12 tadpoles were measured at a time. During the collection period, nobody was allowed to enter the room in order to avoid any disturbance of the animals (minimal noise and visual disturbances). After the hour-long sample collection period, specimens were removed and the water sample was collected. For each sampling batch, we ran a control water sample (i.e. no larvae) to control for potential background hormonal traces in the water. Control samples were pooled and ran on each plate in duplicate. As gently as possible, the specimen was transferred to a 250 mL glass beaker and anaesthetized in 50 mL of 2 g/L tricaine methanesulfonate (MS-222, Ethyl 3-aminobenzoate methanesulfonate; Sigma-Aldrich), dissolved in buffered ultrapure water (Ramlochansingh *et al.,* 2014) until they did not respond to external stimuli. Applying the anaesthetic might be a source of distortion between both WB and tissue CORT measures as MS-222 may affect tissue CORT levels (e.g. Archard and Goldsmith, 2010; Hernández *et al.,* 2012; Smith *et al.,* 2015). Nevertheless, an association between the two measures despite the MS-222 consequently represents a conservative test of congruence. Thus, we opted for the use of the anaesthetic for ethical reasons. We then quickly determined dry blotted body mass in each specimen to the nearest 0.001 g using an electronic balance (Sartorius, Germany). Then, the specimen was immediately placed in a sterile 1.5 mL tube and then snap frozen in liquid nitrogen for tissue CORT analyses. Frozen samples were stored at −80°C until tissue CORT processing. Samples were taken between 1800 and 2100 h.

We stored all water samples at −20°C, by the end of the experiment, we further processed them in a random order during the next month. Thawed samples were first filtered with Q8 Whatman filter paper to remove suspended particles as well as faeces, and then filtered through C18 solid-phase extraction columns (Oasis Vac Cartridge HLB 3 cc/60 mg, 30 μm; Waters, Inc., Switzerland) with a vacuum manifold (Visiprep Vacuum Manifold; Sigma-Aldrich, Germany). We cleaned the manifold before each use with 4 mL of HPLC-grade ethanol and 4 mL of nanopure water. The columns were returned to the −20°C freezer until hormones were eluted with 4 mL of HPLC-grade methanol with a vacuum manifold (Visiprep Vacuum Manifold; Sigma-Aldrich, Germany), which was cleaned with ethanol and nanopure water between batches again. During this process samples were transferred into 5 mL Eppendorf tubes. Afterwards the methanol was evaporated using a sample concentrator (Stuart sample concentrator, SBHCONC/1; Cole-Parmer, UK) under a fine N_2_ stream at 45°C using a block heater (Stuart block heater, SBH130D/3; Cole-Parmer, UK). Dried samples were stored at −20°C until Enzyme-Immunoassay analysis (EIA). We re-suspended the dried sample in a total volume of 125 μL consisting of 5% ethanol (95% lab grade) and 95% EIA buffer. After re-suspension, samples were frozen at −20°C until hormonal level measurements via EIA.

#### Tissue CORT

Tissue CORT samples were shipped on dry ice via overnight express delivery to Doñana Biological Station in Seville, Spain. For extraction, samples were randomly thawed. Whole specimens (Gosner stage 20 and 25) and dissected livers (Gosner stage 30, 37, 39, 42, 46 and 46 + 10 days) were weighed to the nearest 0.0001 g and individually homogenized in 16 × 100 mm glass tubes with 500 μL PBS buffer (AppliChem Panreac, Germany) using a homogenizer at ~17 000 rpm (Miccra D-1, Germany). The tissue blender was washed into the tube with 500 μL PBS buffer, in order to collect the sample residue. Then, it was cleaned with ddH_2_0, and 96% EtOH between samples. After homogenization, 4 mL of a 30%: 70% petroleum ether: diethyl ether dissolvent mixture (both from Sigma-Aldrich, Germany) was added to each sample. Then, samples were vortexed for 60 s and subsequently centrifuged at 1800 g and 4°C temperature for 15 min. After centrifugation, samples were snap frozen in a dry ice ethanol bath for 5 min. The resulting top organic layer containing CORT was collected and placed in a new 13 × 100 mm glass tube. All steps after homogenization were repeated in each sample to ensure maximum CORT extraction. Both ether fractions of each sample were then pooled into a single tube and thereafter evaporated in a sample concentrator (Techne FSC400D; Barloworld Scientific, UK) using a constant but gentle nitrogen flow. Lipids were then resuspended in 315 μL EIA buffer (Assay buffer, DetectX Corticosterone ELISA kit, K014-H5, Arbor Assays, Ann Arbor, MI, USA) using the vortex, and incubated overnight at 4°C.

### CORT assays

The hormonal levels were measured using DetectX Corticosterone ELISA (Enzyme Immunoassay) kits purchased from Arbor Assays (K014-H5, Ann Arbor, MI, USA). This assay has been previously validated for wood frogs (*L. sylvaticus*; Gavel *et al.,* 2019) and has also been successfully used for *Rana arvalis* (Mausbach *et al.,* 2022) and *R. temporaria* (Burraco *et al.,* 2017; Ruthsatz *et al.,* 2023). The manufacturer indicates very low cross-reactivity of this kit against cortisol (0.38%) and aldosterone (0.62%). More information on the cross-reactivity can be found in the kit protocol (https://www.arborassays.com/documentation/inserts/K014-H.pdf).

For WB CORT and tissue CORT, we used the 50 and 100 μL assay format for standard preparations and assays, respectively. We measured CORT concentration in duplicates (WB CORT) and triplicates (tissue CORT) for all samples on 96-well plates according to the kit’s instructions. The plates were read with a Tecan Spark® Microplate Reader (Tecan, Switzerland; in Braunschweig) and a multimode microplate reader (MB-580, Heales; in Seville) at 450 nm. In total, we ran seven plates (i.e. three for WB CORT, four for tissue CORT).

We used MyAssays online tools to calculate the hormonal concentration of samples based on calibration standards provided with the DetectX kit (https://www.myassays.com/arbor-assays-corticosterone-enzyme-immunoassay-kit-improved-sensitivity.assay). A new standard curve for calculation of the results was run for each plate. For WB CORT, the mean coefficient of variation of duplicates for all samples was 8.06%. Intraplate variation was overall 17.78% and interplate variation was on average 22.07%. Animal-free control water samples did not contain detectable levels of CORT. For tissue CORT, we kept all the measurements of triplicates with a coefficient of variation lower than or equal to 30.0% or with an absolute difference between mean and median lower than 2.5 pg. For the samples that did not meet those requirements, we discarded the most different value of the triplet. As a positive control, 1 μL of a solution with a concentration of 0.1 mg x L^−1^ CORT diluted in 99 μL EIA buffer that was run in triplicate on each plate was used to calculate intra- and interplate coefficient of variation. The mean coefficient of variation of triplicates across all samples was 19.07%. Each ELISA plate also included a negative control. Intraplate variation was overall 8.28% and interplate variation was on average 24.82%. The average background corticosterone recorded in our negative control samples was subtracted from hormone samples. Average R^2^ for the 4PLC fitting curve was 0.994 for WB CORT and 0.995 for tissue CORT.

### Statistical analyses

As 13 samples got lost during the procedure due to problems during the tissue CORT extraction, we assigned the remaining samples to the following five consecutive ontogenetic groups for analyses: (1) = newly hatched (external gills; Gosner stage 20), (2) pre-metamorphic (hind limb development; Gosner stages 25 and 30), (3) pro-metamorphic (toe separation and forming of hind limb tubercles and subarticular patches; Gosner stages 37 and 39), (4) metamorphic (onset of metamorphic climax, at least one forelimb present; Gosner stage 42) and (5) post-metamorphic (Gosner stage 46, completion of metamorphosis and juvenile froglets, 10 days after completing metamorphosis).

WB CORT was expressed as release rate that is the amount of CORT in the water adjusted for time (i.e. pg x h^−1^; Gabor *et al.,* 2013a). Tissue CORT levels were expressed in pg. As correcting for body size by using body mass may not yield an accurate scaled CORT release rates or levels (Millikin *et al.,* 2019a) due to the non-linear scaling of body mass and body length, we accounted for body mass (WB CORT) or tissue mass (tissue CORT) by including it as a covariate in the models according to McClelland and Woodley (2021).

**Figure 1:**
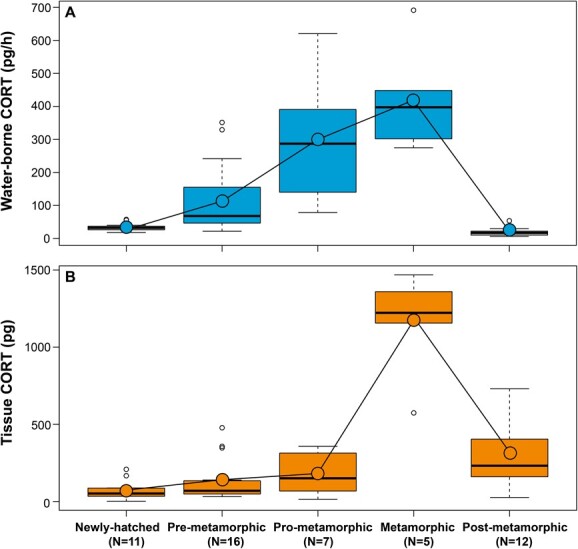
(**A**) WB CORT release rates (pg/h) and (**B**) tissue CORT levels (pg) measured at five ontogenetic stage groups: (1) = newly hatched (external gills; Gosner stage 20), (2) pre-metamorphic (hind limb development; Gosner stages 25 and 30), (3) pro-metamorphic (toe separation and forming of hind limb tubercles and subarticular patches; Gosner stages 37 and 39), (4) metamorphic (onset of metamorphic climax, at least one forelimb present; Gosner stage 42), and (5) post-metamorphic (Gosner stage 46, completion of metamorphosis and juvenile froglets, 10 days after completing metamorphosis). Boxes and whiskers show 25th to 75th and 10th to 90th percentiles, respectively; black lines indicate the median. *N* = individuals sampled at each group. White dots = outliers. Blue and orange dots = mean values at each group. Solid line = cumulative means across ontogeny. See text for further details.

For all statistical tests Cran R (Version 4.1.1, R Development Core Team, 2021) for Windows was used. For all tests and models statistical significance was set at α < 0.05.

#### *CORT levels during development of* R. temporaria

Parametric assumptions were tested using Kolmogorov–Smirnov tests (lillie.test function in the nortest package; Gross *et al.,* 2015) for normality as well as visual inspection of Q-Q plots made with *ggnorm* function. CORT values were log-transformed to fit parametric assumptions. To investigate how both WB and tissue CORT levels change during development of *R. temporaria*, we fitted two linear mixed models to include both fixed and random effects. In both models, we used likelihood ratio tests to determine the significance of each factor. We ran the *lmer* function in the lme4 package (Bates *et al.,* 2007). We included ‘ontogenetic stage group’ as fixed factor. ‘Body mass’ was included as covariate as WB CORT release rate and tissue CORT were not corrected by body mass. To address dependencies in the data, the variable ‘aquarium’ was included as a random factor to all models. Models were followed by post hoc comparisons on adjusted means (lsmeans package; Lenth and Lenth, 2018) with false discovery rate (FDR)-correction to compare all possible pairwise combinations of ontogenetic stage groups.

#### *Validation of WB CORT for developmental stages of* R. temporaria

In order to assess the comparability of the WB CORT and the tissue CORT methods to measure stress, we performed linear regressions on (i) pooled samples from all developmental groups and (ii) samples separated per developmental group.

## Results

### *CORT levels change during development in* R. temporaria

Both WB CORT release rates (F_4,45_ = 34.226; *P* < 0.001) and tissue CORT levels (F_4,43_ = 10.023; *P* < 0.001) changed significantly throughout ontogeny (Fig. 1A,B). Mass covaried with both WB (F_1,45_ = 16.106; *P* < 0.001) and tissue CORT (F_1,43_ = 17.251; *P* < 0.001). For both methods, CORT levels increased with development resulting in the highest mean ± SD CORT levels in tadpoles at the metamorphic stage (WB CORT: 422.75 ± 165.91 pg/h; tissue CORT: 1155.93 ± 347.16 pg). WB CORT release rates were the lowest in post-metamorphic froglets (18.71 ± 12.86 pg/h) followed by newly hatched tadpoles (33.85 ± 12.90 pg/h). Tissue CORT levels were the lowest in newly hatched tadpoles (71.31 ± 64.11 pg/h). WB CORT release rates were lower than tissue CORT levels at all ontogenetic stages.

### Validation

**Figure 2:**
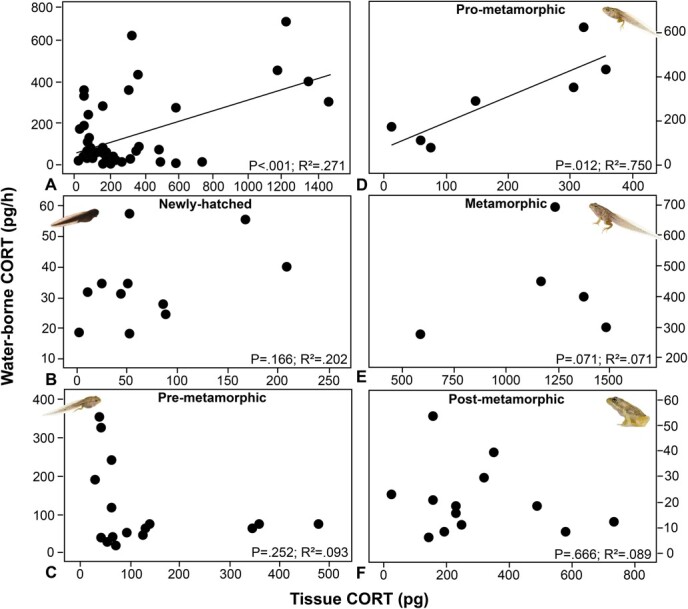
Correlations between tissue CORT and water-borne CORT levels in *R. temporaria* at five different ontogenetic stage groups: (**A**) across ontogeny (*N* = 51), (**B*)*** newly hatched (*N* = 11), (**C**) pre-metamorphic (*N* = 16), (**D**) pro-metamorphic (*N* = 7), (**E**) metamorphic (*N* = 5) and (**F**) post-metamorphic (*N* = 12). Each point is one individual (some points overlap). Regressions lines are presented for significant correlations. Photos of *R. temporaria* by Tim Hunt.

Levels of WB and tissue CORT were correlated when pooling all samples across treatment groups (R^2^ = 0.271; *P* < 0.001; *N* = 51; Fig. 2A). However, when ontogenetic stage groups were analysed separately, only pro-metamorphic tadpoles showed a significant correlation between WB CORT release rates and tissue CORT levels (R^2^ = 0.75; *P* = 0.012; *N* = 51; Fig. 2).

**Figure 3:**
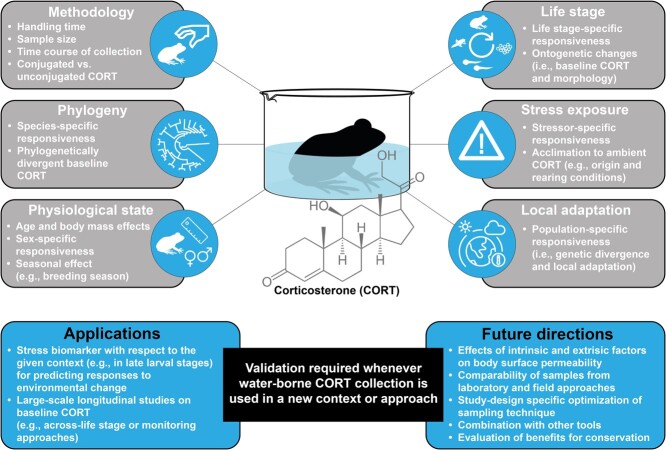
Synthesis of context-dependent variables that might affect the reliability of WB CORT, recommendations and perspectives on the use of this method that will aid in broadening its application as a non-invasive tool in amphibian conservation physiology. See text for further details.

## Discussion

### Non-invasive WB corticosterone assay is a valid tool to assess the physiological state in pro-metamorphic amphibian larvae

Amphibian larvae are considered to be particularly sensitive to environmental stressors due to their limited capacity for habitat selection (Yu *et al.,* 2015) and their highly permeable skin (Hayes *et al.,* 2006). Therefore, non-invasive conservation tools might help to assess the stage-specific impact of environmental stressors on amphibian larvae by allowing for repeated measurements across development. Here, we initially assessed how both tissue and WB CORT change throughout ontogeny and then evaluated whether measuring WB CORT release rates can serve as a valid non-invasive method to determine the physiological state of amphibians in response to environmental stressors at different developmental stages.

As a reliable biomarker, WB CORT release rates would represent the organismal CORT levels of an animal. Here, WB CORT correlated with tissue CORT levels in pro-metamorphic larvae of *R. temporaria*. Our results are in line with findings of a recent study evaluating WB CORT assay at different life stages of the Northern leopard frog (*L. pipiens*) (McClelland and Woodley, 2021) indicating that WB CORT might be a valid method in late-stage larvae of anurans across species. During pro-metamorphosis, endogenous CORT levels are higher than during pre-metamorphosis (present study; Denver, 1998; Chambers *et al.,* 2011; Regueira *et al.,* 2022) and the body surface is still large enough to allow for transdermal CORT release as the skin is low in keratinization and gills are still present (Burggren and West, 1982; Shi, 2000). In contrast, we could not find a correlation of WB and tissue CORT in all other ontogenetic stage groups. This lack of association may be partly due to low statistical power since all data pooled together showed an unequivocal strong correlation between the two measures. However, there are biological reasons for this pattern, as there could be a mismatch between the endogenous CORT production and the surface of skin and gills allowing for transdermal CORT release. In hatchlings and pre-metamorphic larvae, endogenous CORT levels are low, and the transdermal hormone release may therefore be too low to give a reliable signal. In metamorphs, the keratinization of the skin (Nishikawa and Yoshizato, 1986; Nishikawa *et al.,* 1989) and the degeneration of the gills (Burggren and West, 1982) might impair the transdermal release of the hormone.

Based on the results of the present study and a comparable study (McClelland and Woodley, 2021), we conclude that WB CORT sampling is a promising, non-invasive conservation tool for studies on baseline CORT in late-stage anuran larvae (Fig. 3). To overcome the life stage-specific limitations of WB CORT, we suggest studying the life stage-specific CORT release capacity of different tissues such as the skin and gills. Moreover, the amount of CORT released via urine and faeces during the collection period (Gormally and Romero, 2020) might give further insights into the reliability of WB CORT as representative of organismal CORT levels at different life stages. Nevertheless, both collection methods consistently revealed a non-linear pattern of ontogenetic change in CORT and detected the peak in endogenous CORT levels at metamorphic climax and its dramatic decrease after completion of metamorphosis. Therefore, despite the lack of correlation between WB and tissue CORT within different ontogenetic stage groups, our results imply that WB CORT levels are physiologically relevant and thus, biologically meaningful. Consequently, we suggest that WB CORT collection might also be a good tool to be used in comprehensive longitudinal studies such as cross-life stage studies or monitoring approaches (Fig. 3).

### Recommendations and future perspectives on the use of WB CORT as a non-invasive tool in amphibian conservation physiology

With amphibians being at the forefront of biodiversity loss (IUCN, 2023), the use of non-invasive tools that characterize the physiological state and thus, predict the multi-scale responses to environmental change or monitor the success of conservation programs are of increasing interest in amphibian conservation physiology (Madliger *et al.,* 2018). Beside skin secretion (Santymire *et al.,* 2018) and salivary samples (Hammond *et al.,* 2018), WB CORT collection (Gabor *et al.,* 2013a) is increasingly used in eco-evo-physiological studies on amphibians. However, in order to optimize protocols and to guarantee comparability of results between studies, we urge future studies to validate the use of this method whenever it is used in different contexts or approaches. To that end, we present a synthesis on the context-dependent variables that might interfere with the reliability of WB CORT, as well as recommendations and future perspectives aimed at broadening its application as a non-invasive tool in amphibian conservation physiology (Fig. 3):

- Several methodological aspects might affect WB CORT measures. For example, *handling stress* has been shown to induce a short-term increase in whole-body (Glennemeier and Denver, 2002), plasma (Woodley and Lacy, 2010; Bliley and Woodley, 2012) and urinary CORT (Narayan *et al.,* 2012) in amphibians. Gabor *et al.* (2016) showed also an increase in WB CORT release rates in response to handling in adult aquatic salamanders. Albeit a recent study could not confirm an increase in WB CORT following handling in larval amphibians (McClelland and Woodley, 2021), handling time and impact should be minimized before the collection period. Likewise, WB CORT measures might differ with *collection time course* as well as *daytime of collection* and should therefore be standardized among studies. However, whereas most of the studies that resulted in correlated measures between plasma and WB CORT used 1 h containment, a study on adult Túngara frogs (*Engystomops pustulosus*) demonstrated a reliable correlation after longer collection periods (i.e. 2 h; Baugh *et al.,* 2018). Future studies should therefore consider that the optimal collection time course might depend on further variables such as life stage, or the species measured. Furthermore, animals are likely to release urine or faeces into the holding water to different extent that may affect the ultimate CORT concentrations since antibodies in well-established EIA kits do not specify whether the measured CORT is conjugated (i.e. from urine or faeces) or unconjugated (i.e. waterborne CORT), thereby measuring total CORT (McClelland and Woodley, 2021). Since measuring free or unconjugated CORT is advantageous as it is the physiologically active form (Ellis *et al.,* 2013; McClelland and Woodley, 2021), we recommend filtering the holding water after collection period or taking advantage of different antibodies for detecting different CORT metabolites. To the best of our knowledge, only one study so far has determined different CORT metabolites from holding water in amphibians (Nagel *et al.,* 2019). Finally, CORT samples exhibit considerable variability, making it difficult to discern connections between stressors and WB CORT. Inconclusive results can be avoided by using a large sample size.
- Despite the increasing evidence that this method is a valid indicator for tissue content of this hormone in the species studied (e.g. present study: *R. temporaria*; Gabor *et al.,* 2013a: *Eurycea nana* and *Alytes obstetricans*; McClelland and Woodley, 2021: *L. pipiens*; Forsburg *et al.,* 2019: *R. berlandieri*; Davis *et al.,* 2020: *Alytes obstericans*; Ledón-Rettig *et al.,* 2023: *Spea bombifrons*), baseline and stress induced CORT levels are known to be phylogenetically divergent (Kulkarni *et al.*, 2017) and thus, the reliability of WB CORT needs to be validated for each species. For example, Gabor *et al.* (2016) found that WB CORT release rates differed between three salamander species of the genus *Eurycea* (*E. nana*, *E. sosorum*, *E. tonkawae*). Moreover, Tornabene *et al.* (2021) found that WB CORT served as a biomarker of salt stress only in *L. pipiens* but not in boreal chorus frogs (*Pseudacris maculata*) or barred tiger salamanders (*Ambystoma mavortium*). In contrast, Millikin *et al.* (2019a) could not validate the method for adult spotted salamanders (*Ambystoma maculatum*).
- Glucocorticoids are directly linked with the physiological state of an organism as they aid in the regulation of metabolic, immune, behavioural, and reproductive function (Moore and Jessop, 2003; Crespi *et al.,* 2013; Rollins-Smith, 2017) and therefore might underly not only ontogenetic but also seasonal fluctuations. For instance, Nagel *et al.* (2019) found that CORT levels of the aquatic salamander *Necturus beyeri* reveal a seasonal pattern linked to reproduction. CORT levels might further change with individual age and body size (i.e. SVL, body mass) as well as body condition (Ruthsatz *et al.,* 2023). Such individual variation might incur difficulties when comparing WB CORT measures (Scott and Ellis, 2007). Millikin *et al.* (2019b), for example, demonstrated that WB CORT release rate was associated with salamander body size even after correction for body size. Therefore, considering body condition index (MacCracken and Stebbings, 2012) or surface-area-to-volume ratio (Ferreira Amado *et al.,* 2019) might help to overcome inconsistent results when controlling for variation in body size using SVL or body mass (Millikin *et al.,* 2019a). Comprehensive longitudinal studies such as across-life stages ones or monitoring approaches aiming to use WB CORT as a tool need to address possible seasonal fluctuations as well as changing physiological states over time.
- Endogenous CORT levels change throughout ontogeny with a peak at metamorphic climax followed by a dramatic decrease after completion of metamorphosis in juveniles (Denver, 1998; Chambers *et al.,* 2011; Regueira *et al.,* 2022). As demonstrated in the present study, WB CORT release rate can reflect this ontogenetic change in endogenous CORT levels. However, life stage might affect CORT release rates through the skin as the amphibian skin changes dramatically during development and metamorphosis (Shi, 2000; Tata, 2006). Similarly, McClelland and Woodley (2021) validated WB CORT as a technique for pro-metamorphic tadpoles but not for pre-metamorphic tadpoles, tadpoles during metamorphosis, or metamorphs of the Northern leopard frog (*L. pipiens*). Contrary, Millikin *et al.* (2019a) could not find correlations between plasma and WB CORT levels in any life stage group of spotted salamanders (i.e. larvae, metamorphs, adults). These correlations, however, might be species-specific and should be validated accordingly.
- In an growing number of experiments, WB CORT has been successfully validated as an indicator for measuring a response to acute stress exposure (e.g. Novarro *et al.,* 2018: temperature; Shidemantle *et al.,* 2022: salinity; Ruthsatz *et al.,* 2023: nitrate contamination; Davis *et al.,* 2020: ranavirus infection and agricultural pollutants; Gabor *et al.,* 2018: urbanization; Barnhart *et al.,* 2020: *Batrachochytrium salamandrivorans* infection; Forsburg *et al.,* 2021: light pollution; Gabor *et al.,* 2013b, 2015: *Batrachochytrium dendrobatidis* infection). However, reliability of WB CORT might differ between different stressors or certain species might respond differently to a stressor for that WB CORT has already been validated. For example, Tornabene *et al.* (2021) found that WB CORT is no reliable indicator for endogenous CORT levels in response to increased salinity in two out of three tested species. Similarly, exposure to certain stressors that affect the skin or gill surface such as aquatic contaminants or pathogens (Fenoglio *et al.,* 2009; Bernabò *et al.,* 2013; Strong *et al.,* 2016), might impair the CORT release and so, WB CORT might be less reliable in detecting fluctuations in organismal CORT. Future studies should therefore assess the impact of intrinsic and extrinsic factors on body surface permeability. Likewise, the specific rearing conditions might affect CORT release rates because tadpoles can take up the released CORT of their conspecifics in the same tank through their skin and gills (Wack *et al.,* 2010; Gabor *et al.,* 2018; Forsburg *et al.,* 2019). This ambient CORT might influence the HPI axis, influencing CORT release rates and causing confounding effects on CORT release rates (Tornabene *et al.,* 2021). For independent WB CORT measures, individual housing of the tadpoles would be advantageous even if is often not feasible in common garden experiments.
- There is increasing evidence that populations can acclimate or evolve to human-caused environmental change by exhibiting increased tolerance to stressful environments. The mechanisms that allow for such local adaptations may affect baseline CORT levels as well as the responsiveness to acute stress (Telemeco and Addis, 2014; Novarro *et al.,* 2018). Consequently, CORT levels might differ between more tolerant and less tolerant populations (Rich and Romero, 2005; Narayan and Hero, 2014) influencing the reliability of WB CORT as an indicator of disturbance across populations that vary in tolerance to anthropogenic stressors. For example, Shidemantle *et al.* (2022) found that populations of *L. sylvaticus* with different levels of salinity tolerance show variation in baseline and stress-induced CORT but conclude that WB CORT still represents a viable indicator of condition. Similarly, in spring salamanders (*Gyrinophilus porphyriticus*) living with fish predators, Bryant *et al.* (2022) measured lower WB CORT release rates than in those populations without fish predators indicating that chronic predator presence reduces glucocorticoid production in response to predators. Also, Mausbach *et al.* (2022) found clear differences in CORT levels among populations of the moor frog (*R. arvalis*) due to genetic divergences and local adaptation. Therefore, WB CORT release rates in response to a specific stressor might be highly population-specific.

### Future perspectives and concluding remarks

Stress physiology measures are among the most commonly used tools in conservation science with GCs being a particularly active field of tool development and refinement (Madliger *et al.,* 2018). Our effort highlights the context-dependent limits and benefits of WB CORT collection as a non-invasive tool in amphibian conservation physiology. We suggest that rather than replacing invasive collection methods, WB CORT complements the tools available for assessing the physiological state of amphibians. This non-invasive tool has the added benefit of allowing for repeatable measurements, which facilitates long-term monitoring of natural populations. We therefore encourage future studies to evaluate this sampling technique under field settings in order to include it into the effective implementation of conservation programs (Gabor *et al.,* 2016; Millikin *et al.,* 2019b; Davis *et al.,* 2020). A meta-analysis summarizing existing research and identifying patterns of GCs as biomarkers for responses to environmental change in amphibians assessed with different tools in both laboratory and field approaches could be a first step in this direction and might also reveal new promising research avenues for future environmental and conservation studies.
